# Supplementary material for: Trustworthiness of information sources on vaccines for COVID-19 prevention among Brazilians
Source: PLoS One. 2023 Jan 3;18(1):e0279393. doi: 10.1371/journal.pone.0279393 (PMC9810184; doi:10.1371/journal.pone.0279393)
Supplement: Attachment — Submitted filename: Review comments on PONE.docx [file pone.0279393.s001.docx]

Review comments on “PONE-D-21-27720”

This is an important, timely piece of research, but its effort seems to fall short of what it intends to tackle due to the following reasons to be addressed below. This paper can be remedied to some extent if the original survey questionnaire contains the variables needed.

Major issues:

First, the group of authors appears to have used the concepts of reliability and trust (or trustworthiness) interchangeably. But scholars have made effort to distinguish between them. I personally think trustworthiness is the correct and appropriate one to be adopted, instead of reliability.

Second, communication researchers and news outlets (e.g., *The Economist*) have suggested strongly that political stance (or party identification) has played an underlying role in individuals’ attitudes toward vaccination against the COVID-19 pandemic, notably, in the United States. But the authors have ignored this crucial variable in examining this issue. If the survey questionnaire does contain this variable, it is strongly urged that the authors should include it for revised analysis.

Third, the authors should have provided the concrete operationalization of the key concepts, particularly of the “hesitant vs. non-hesitant” variable. Another way is to list the 27-items questionnaire as an appendix for review evaluation.

Fourth, information sources (e.g., scientific papers, newspapers, TV, or friends/relatives as interpersonal communication) can be differentiated from news sources (e.g., doctors, minister of health, health professionals). The entangled use of the two distinguishable groups of sources can severely undermine the validity of the study. For one, as the authors themselves pointed out (on page 13) that most people usually would not read scientific papers, this operational definition of the concept would be very inappropriate. Neither would most individuals actively access the WHO’s information on the web, though they might have access to doctors when they visited medical facilities. By and large, some key sources should have been operationalized as news sources, who—trusted or not—would offer their viewpoints and discussions through news media as information sources.

Fifth, the “TREND study (page 3)” of the assessment of vaccine hesitancy appears not comparable with what has been done because the survey collected data for only one week.

Sixth, what are the three open-ended questions? Their use and explications would have offered more insight for this issue.

Minor issues:

First, on page 3, the news media have indeed long been deemed as a non-trustful institute, as opposed to the claim by the authors.

Second, the valid sample size should have been listed earlier but was not given until page 6.

Third, allowing respondents to review and change their answers may not be a good idea as it could contaminate the study, undermining its validity.

Fourth, is income (variable) monthly? And the terms (page. 6) used (e.g., 5 minimum wages) are not familiar to international readers.

Fifth, there are several unclear or awkward statements that need to be rewritten or clarified. On page 8, “However, we found a difference between the hesitant… and the Ministry of Health…” On page 9, “This same variation depending… Newspapers, and doctors.” Moreover, on page 14, the first complete paragraph was confusing and needs clarification. And what is the sign of the strong correlation mentioned?

Sixth, a contradiction occurs on pages 8 and 11, concerning the role of gender (female in p. 8 vs. male, in p. 11) in the logistic regression model.

Seventh, on page 12, how frequently the turnover of health ministers has been? Please briefly state it.

Eighth, also on page 12, although citing a study, the statement, “… distrust in the WHO… to reject any proposal from these sources,” sounds like a second guess. Any more concrete evidence to support the claim?

Ninth, at the bottom of page 13, TV news does disseminate information very quickly, but it is usually quite superficial, as opposed to the claim of being comprehensive, unless it was an unusual in-depth piece.

Tenth, the use of the word “global” in this context is not very proper as it appears to mean “overall,” rather than international.
