# Supplementary material for: Trustworthiness of information sources on vaccines for COVID-19 prevention among Brazilians
Source: PLoS One. 2023 Jan 3;18(1):e0279393. doi: 10.1371/journal.pone.0279393 (PMC9810184; doi:10.1371/journal.pone.0279393)
Supplement: Attachment — Submitted filename: Response to Reviewers.docx [file pone.0279393.s003.docx]

**Response to Reviewers**

Responses to points raised by the academic editor and reviewers of PLOS ONE [PONE-D-21-27720R1]

**Trustworthiness of information sources on vaccines for COVID-19 prevention among Brazilians**

**Reviewer 1**

Question 1: The author misconceived my question about the “TREND study.” My question about it simply is: Why a one-week study can be called a trend study, which literally should have lasted some time during which a certain trend surfaces?

Answer 1: TREND is an acronym for the title of the original study in Portuguese. The intent of the initial study was to carry out recruitment over a year and for this reason the acronym seemed appropriate. However, the study had great repercussion already in the first week of recruitment and it was not necessary to extend the study any longer. The study name has been removed to avoid confusion.

Question 2: The use of some variables and their operations appear inconsistent in method and results sections, i.e., schooling, which seems to have a varying operational definition. More specifically, “secondary” in method section appears to be “high school” in results section; use of secondary is less suitable. Moreover, the differentiation between “somewhat afraid” and “more or less afraid” for “fear of catching COVID-19” variable is confusing and hard to discern.

Answer 2: Data from schooling was adjusted: “schooling (primary or less, incomplete high school, complete high school, incomplete university degree or complete university degree)”

The difficulty may be in the translation. I'm changing "somewhat afraid" to "a little afraid" to improve understanding. In addition, when we prepared the questionnaire, the options were presented so that the participant could understand that there was a hierarchical order of fear in contracting covid-19

Question 3: Also concerning methods, the sampling method appears to be snowballing, a voluntary, non-randomized, and non-representative sampling technique. As such, the traditional statistical analysis, p-value decision-making and the ensuing interpretation should be conducted more conservatively.

Answer 3: The statistical analysis was performed considering nonparametric two-tailed tests, which consider the empirical distribution of the observed data. The interpretations were always made in light of the fact that this was a convenience sample, with a strong bias towards individuals who have access to technology in the Brazilian population. The sampling method was the "virtual snowball", which started by sending invitations with the link to access the electronic questionnaire.

Question 4: Methods/results still:

(A) vaccine efficacy is questioned about “importance” in methods, but it was presented as “understandability” in results. Why so?

Answer A: We apologize for the error in the methods section. The correct one is "Opinion about the released data on the effectiveness of vaccines (very understandable, understandable, don't know, confusing, very confusing) "

(B) Residence in state capital as a potential key variable in methods is not specifically mentioned in results section.

Answer B: We will add in the results section the information about participants' residence in the state capital. "Among the participants, 107,141 (61.87%) resided in the state capital.

Question 5: I am curious about why schooling is not a factor in the relatively greater trust of ministry of health by the non-hesitant.

Answer 5: The ministry of health communicated in a confused way during the pandemic due to several changes of ministers (four changes), and aligned itself with the denialist of science encouraged by the president of the republic. We believe that political views were more linked to trust in the ministry of health among those who were hesitant. As the political views were not addressed, this was not taken into the discussion as they are just speculations. Current president Jair Bolsonaro represents the far right ideology that has the support of the richest that usually have higher degree of education.

Question 6: I would recommend adding a cross-tab table/chi-square test between “hesitancy” and “strategy” and discussing its outcomes, which may come up with something interesting.

Answer 6: figure 4 adress this recommendation

Question 7: Change all wordings of reliable/reliability to truthful/ truthfulness/ trustworthiness (i.e. in page 2) throughout the manuscript as you have adopted my previous suggestion on use of the concept.

Answer 7: The changes were made.

Question 8: Do not use “unbiased press”; just write “the press,” which is outright neutral.

Answer 8: The changes were made.

Question 9: Is National Immunization Program (NIP, instead of PNI) (page 3)?

Answer 9: The changes were made.

Question 10: Quickly provide the total number of valid cases (173,178) at the end of the “Ethical aspects,” so the readers can have an immediate sense about it. Also, the figure/percentage of hesitant vs. non-hesitant has never physically been mentioned in the text. Please provide it in the early part of the results section for easy reading.

Answer 10: The total number of valid cases (173,178) will be added at the end of the "Ethical Aspects".

In figure 2, we detail how many were hesitant and non-hesitant (18,250 hesitant and 154,928 non-hesitant). The percentage of hesitant versus not hesitant was mentioned at the beginning of the results.

Question 11: Is the income in results monthly? If so, write monthly income.

Answer 11: It was corrected to monthly income.

Question 12: What are the increase/decrease in odds ratio represented by dark/light gray compared to?

Answer 12: Table 1. Multivariate logistic regression to assess the factors associated with trust in the information source. The dark gray cells represent an increase in the odds ratio (OR ≥1,1) and the light gray cells a decrease in this odds ratio (OR <1) with respect to trust in the information source.

Question 13: The statement from lines 432 to 434 is unclear. Please clarify it.

Answer 13: It was added “Thus, information about the progress of the pandemic could only be obtained through these vehicles, which included one of the most watched television channels in Brazil.”

Question 14: Explain further the claim on the effectiveness of adopting behavioral economics in motivating behavioral change concerning vaccination. (lines 474 ~ 476).

Answer 14: The sentence was changed to: “Seeking alternatives, a study suggests that using strategies as those that motivate behavioral change, like nudges, through the architecture of choice could enhance vaccine aceptance[26].”

Question 15: I don’t see figure 4.

Answer 15: Figure 4 "Indicated strategies to increase the percentage of vaccine adherence to prevent COVID-19 according to intention to vaccinate" was mentioned in the text, in the section "Strategies to increase vaccine adherence" and attached at the end of the submitted file.

Question 16: Change p< .000(0) to p < .001 or p< .0001

Answer 16: The changes were done.
